# Roles of intercellular cell adhesion molecule-1 (ICAM-1) in colorectal cancer: expression, functions, prognosis, tumorigenesis, polymorphisms and therapeutic implications

**DOI:** 10.3389/fonc.2022.1052672

**Published:** 2022-11-23

**Authors:** Zhiyuan Qiu, Yan Wang, Zhao Zhang, Rong Qin, Yong Peng, Weifeng Tang, Yan Xi, Guangyu Tian, Yeqing Zhang

**Affiliations:** ^1^ Department of Oncology, the Affiliated People’s Hospital of Jiangsu University, Zhenjiang, Jiangsu, China; ^2^ Department of Cardiothoracic Surgery, Nanjing Drum Tower Hospital, Nanjing, Jiangsu, China; ^3^ Department of Geriatrics, the Affiliated People’s Hospital of Jiangsu University, Zhenjiang, Jiangsu, China; ^4^ Department of Oncology, Jiangdu People’s Hospital Affiliated to Medical College of Yangzhou University, Yangzhou, Jiangsu, China; ^5^ Department of Vascular Surgery, the Second Affiliated Hospital of Soochow University, Suzhou, Jiangsu, China

**Keywords:** ICAM-1, colorectal cancer, polymorphisms, prognosis, tumorigenesis

## Abstract

Colorectal cancer (CRC) is a major global health problem and one of the major causes of cancer-related death worldwide. It is very important to understand the pathogenesis of CRC for early diagnosis, prevention strategies and identification of new therapeutic targets. Intercellular adhesion molecule-1 (ICAM-1, CD54) displays an important role in the the pathogenesis of CRC. It is a cell surface glycoprotein of the immunoglobulin (Ig) superfamily and plays an essential role in cell-cell, cell-extracellular matrix interaction, cell signaling and immune process. It is also expressed by tumor cells and modulates their functions, including apoptosis, cell motility, invasion and angiogenesis. The interaction between ICAM-1 and its ligand may facilitate adhesion of tumor cells to the vascular endothelium and subsequently in the promotion of metastasis. ICAM-1 expression determines malignant potential of cancer. In this review, we will discuss the expression, function, prognosis, tumorigenesis, polymorphisms and therapeutic implications of ICAM-1 in CRC.

## Introduction

Colorectal cancer (CRC) with a particularly high prevalence in China is a major health problem and one of the major causes of cancer-related death worldwide (1, 2). In recent years, both the morbidity and mortality of CRC have increased (1), thus it is very important to understand the pathogenesis of CRC for early diagnosis, prevention strategies and identification of new therapeutic targets. Some studies have focused on the identification of biomarkers in CRC, taking cell adhesion molecules (CAMs) as their subject.

CAMs refer to those cell surface structures that allow cells to adhere to each other and the extracellular matrix. They are critical regulators of cellular homeostasis and function, and play crucial roles in tumorigenesis, progression and metastasis (3, 4). Intercellular adhesion molecule-1(ICAM-1, CD54) is one of CAMs, which displays an important role in the the pathogenesis of CRC. In this review, we will discuss the expression, function, prognosis, tumorigenesis, polymorphisms and therapeutic implications of ICAM-1 in CRC.

## ICAM-1 structure, expression and function

ICAM-1, located on chromosome 19p13, is a cell surface glycoprotein of the immunoglobulin (Ig) superfamily of CAMs, and consists of 5 extracellular Ig-like domains, a transmembrane domain and a short cytoplasmic tail (5). The Ig-like domains mediate ICAM-1 interactions with its two major ligands, macrophage-1 antigen (Mac-1, CD11b/CD18) and lymphocyte function-associated antigen-1 (LFA-1, CD11a/CD18) (6).

ICAM-1 is expressed in various cell types (epithelial cells, keratinocytes, fibroblasts and immune cells) and plays an essential role in cell-cell, cell-extracellular matrix interaction, cell signaling and immune process (7, 8). It serves as a biosensor to transducer outside-in-signaling *via* association of its cytoplasmic domain with the actin cytoskeleton following ligand engagement of the extracellular domain. Upon ligation, ICAM-1 undergoes dimerization and clustering through homotypic binding between Ig domains (7, 8).

Epithelial cells (ECs) of normal human colon do not express ICAM-1, but it can be expressed subsequent to malignant transformation. It has also been shown that ICAM-1 is related to the mesothelial adhesion, malignant potential, occurrence and progression of CRC (9–12). The interaction between ICAM-1 and its ligand may facilitate adhesion of tumor cells to the vascular endothelium and promote metastasis subsequently. The patients with increased ICAM-1 expression have more advanced stage, as it promotes the tumor growth (11, 13). Its expression was also associated with the cell differentiation of CRC. Higher ICAM-1 expression was found in better differentiated CRC cells compared to lower ICAM-1 expression in poorly differentiated CRC cells, which demonstrated that ICAM-1 promoted CRC differentiation and retarded metastais (14). ICAM-1 may play an important role in the immune response. The increased ICAM-1 expression might reflect the elevated immunity against tumor cells and ICAM-1 renders tumor cells more sensitive to lymphocyte-mediated lysis (15).

## The prognostic significance of ICAM-1 expression in CRC

The prognostic significance of ICAM-1 expression remains controversial in CRC. ICAM-1 plays a dual role in CRC, and its impact depends on whether this protein is expressed in a membrane-bound or a soluble form (16–20). In CRC, the increased expression of membrane-bound ICAM-1 was associated with the favorable prognosis (16, 21). Maeda K et al. reported a better prognosis for CRC patients with membrane-bound ICAM1-positive (16). Tachimori A et al. also reported that an increased membrane-bound ICAM-1 expression inhibited the tumour growth and was correlated with a favorable prognosis in CRC (22). Leqi Zhou et al. and Mlecnik B et al. reported that a high expression of ICAM-1 was relevant to a prolonged survival (21, 23).

The favorable prognosis may be attributed to two mechanisms: one is that ICAM-1 may play an important role in the immunosurveillance and enhances lymphocyte-mediated cytotoxicity (15, 24–26). T cells are important for killing tumor cells and the increased ICAM-1 expression on CD8^+^ T cells activates the antitumor function of CD8^+^ T cells. ICAM-1 expressed by tumor cells may lead to T cell-specific recognition and enhanced T cell adhesion (27). Tachimori et al. showed that more lymphocytes adhered to CRC cells when ICAM-1 expression was upregulated (22). The other potential mechanism is that ICAM-1 may play an important role in the tumor microenvironment (TME). Upregulation of ICAM-1 in CRC cells could increase cytotoxic lymphocytes (CTLs) infiltration and the expression of cytolytic immune effector molecules in the TME, which is associated with favorable prognosis in CRC (4, 28–30). Increased CTLs was observed in the TME of ICAM-1 positive CRC compared to that of ICAM-1 negative CRC (13). Fisher et al. showed that ICAM-1 blockade decreased CTLs infiltration in the TME (31). The increased ICAM-1 expression on other cells in the TME also enhances the tumor infiltration and function of CTLs (23, 32, 33).

However, in some reports, the ICAM-1 expression was correlated with a worse prognosis (34, 35). For instance, Ionescu C et al. reported overexpression of ICAM-1 was correlated with lower overall survial (OS) (34). There is no clear explanation for the apparently contrary roles of ICAM-1, suggesting that the function of ICAM-1 is context dependent and modulated by the action of other membrane receptors.

## Factors regulating ICAM-1 expression

**Figure 1:**
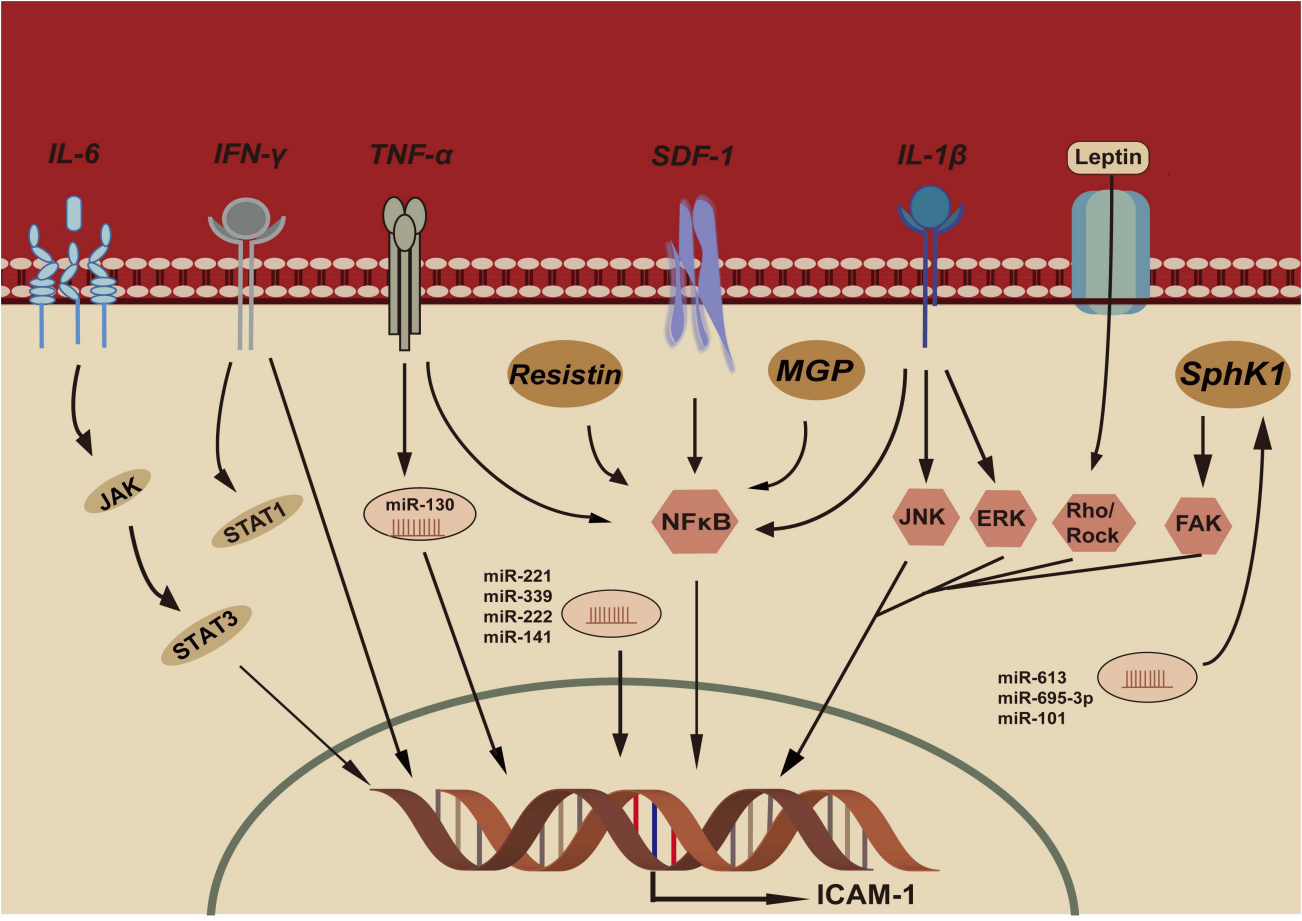
IL-6, Interleukin-6; IFN-γ, Interferon- γ; TNF-α, Tumor necrosis factor- α; SDF-1, Stromal cell derived factor-1; IL-β, Interleukin- β; MGP, Matrix gla protein; SphK1, Sphingosine kinase-1; JAK, Janus kinase; STAT3, Signal transducer and activator of transcription 3; STAT1, Signal transducer and activator of transcription 1; NFκB, NFκB pathway; JNK:c-Jun N-terminal kinase pathway; ERK, Extracellular signal-regulated kinase pathway; FAK, Focal adhesion kinase pathway; Rho/Rock, Rho/Rho-associated kinase pathway; ICAM-1, Intercellular adhesion molecule-1.

Several studies have focused on the factors regulating ICAM-1 expression, and the mechanisms seem to be multiple. **Figure 1** demonstrates the potential mechanisms of ICAM-1 expression in CRC.

## Cytokines

A variety of cytokines may regulate ICAM-1 expression, such as TNFα, IFNγ and IL-1β (36, 37). The regulatory mechanism is mainly involved in activating signal pathways through the binding on the ICAM-1 promoter.

## Tumor necrosis factor

TNF-α can regulate ICAM‐1 expression, which results in enhanced lymphocytic infiltration and tumor apoptosis (36). TNF-α response element is important in regulating ICAM-1 expression. This response element possesses an NF-κB binding site and NF-κB plays a significant role in TNF-α induced ICAM-1 expression (38).

## Interferon-γ

IFNγ can also induce ICAM-1 expression and STAT-1 (signal transduction and activator of transcription) protein is upregulated during IFN-γ response (39). The IFN-γ response element (IRE) plays a important role in ICAM-1 expression (40). Upon IFN-γ stimulation, IRE forms a binding complex (IRE-BC) with nuclear proteins, which is required for induction of ICAM-1 expression.

## Interleukin-6

IL-6 is a T-cell-derived cytokine that induces maturation of B cells. IL-6 plays a critical role in metastasis of cancer cells by modulating ICAM-1 expression (40). CRC patients exhibited high level of IL-6 and IL-6 induces ICAM-1 expression *via* IL-6 receptor. JAK-STAT3 pathway and the AP-1 binding site of ICAM-1 are involved in IL-6 mediated ICAM-1 expression (41).

## IL-1β

IL-1β has been reported to induce ICAM-1 expression and is involved in multiple immune and inflammatory responses (42). IL-1β also activates NF-κB pathway, ERK pathway and JNK pathway for ICAM-1 expression.

## Resistin

Resistin is an adipose tissue-secreted form and could also be expressed in peripheral blood mononuclear cells, macrophages, and bone marrow cells (43). Resistin exerts its biological effects by binding to Toll-like receptor 4 (TLR4) and NF-κB can be activated by TLR4 which leads to ICAM-1 expression (44, 45).

## Transcription factors

The expression of ICAM-1 may be regulated by a few transcription factors, such as stromal cell derived factor-1 (SDF-1), Leptin, C/EBPβ (36).

## SDF-1

Abnormal expression of SDF-1 has been detected in CRC and ICAM-1 expression was up-regulated by SDF-1 (46). MAPKs pathway may be involved in the SDF-1-mediated expression of ICAM-1 (47). By MAPKs pathways, SDF-1 activates NF-κB and C/EBPβ to bind to the promoter of ICAM-1, thus leading to ICAM-1 up-regulation in CRC cells (46).

## Leptin

Leptin can induce ICAM-1 expression and the Rho/ROCK (Rho-associated coiled-coil-forming protein kinase, ROCK) pathway may be involved in the leptin-mediated expression of ICAM-1 (48). Z Dong et al. revealed that leptin can induce ICAM-1 expression by the Rho/ROCK pathway (49).

## Sphingosine kinase 1

SphK1 is an oncogene and is associated with angiogenesis, anti-apoptosis and survival of tumor cells (50). The SphK1 expression is enhanced in CRC and enhanced the ICAM-1expression by regulating the FAK pathway in CRC cells (51, 52). The ICAM-1 expression is upregulated with the overexpression of SphK1 and downregulated with the suppression of SphK1 in CRC cells.

## Matrix Gla protein

MGP is a secreted, calcium-binding matrix protein. Overexpressed MGP could be found in CRC, and it may be associated with tumor progression and invasion. Li X et al. revealed that MGP expression increased in CRC and MGP promoted the phosphorylation of NF-κB by upregulating intracellular free calcium concentrations, activating the expression of ICAM-1 (53).

## MicroRNAs

MicroRNAs (miRNAs) also play an important role in regulating the ICAM-1 expression in CRC. Recent studies indicate that ICAM-1 is a direct target of miRNAs, and these miRNAs bind to the untranslated region (UTR) of ICAM-1 and regulate ICAM-1 expression. Mir-221 binded to 3’UTR of ICAM-1 mRNA which resulted in transcription suppression of IFN-γ induced ICAM-1 expression (54). In addition, miR-222 and miR-339 have also been shown to bind 3’UTR of ICAM-1 promoter to suppress the ICAM-1 expression and promoted resistance of cancer cells to CTLs (55, 56). MiR-130 was induced by TNFα and lead to the increased ICAM-1 expression (57). MiR-141 binded to the 3’UTR of ICAM-1 directly and inhibited TNF-α induced ICAM-1 expression in ECs (58). Some miRNAs modulate ICAM-1 expression through down-regulating SphK1 expression in CRC cells, such as miR-613, miR-659-3p, miR-101. These miRNAs targeted SphK1 and downregulated ICAM-1 expression (59–62). SphK1 was an important target of miR-101, and miR-101 down-regulated SphK1 to inhibit ICAM-1 expression in CRC cells (62).

## Genetic variations

Genetic variations in the ICAM-1 gene can regulate the protein expression in various diseases. ICAM-1 rs5498 may affect the expression of ICAM-1 in CRC patients. Wang MY et al. reported that ICAM-1 rs5498 may affect the level of sICAM-1 (63). The level of sICAM-1 at ICAM-1 5498 allele locus in K individuals was higher than that at non-K allele. Wang QL et al. also reported patients with KK genotype showed an increased ICAM-1 expression in CRC and ICAM-1 expression was higher in patients with KK genotype than that with KE+EE genotypes (64). ICAM-1 5498 is a non-synonymous mutation, which leads to the increased expression of ICAM-1 and affects the function of ICAM-1.

## ICAM-1 expression and metastasis in CRC

ICAM-1 expression may decrease CRC metastasis. In CRC, the high expression of membrane-bound ICAM-1 was associated with a lower incidence of liver and lymph node metastases (14, 16, 17). The upregulation of ICAM-1 inhibited tumor metastasis in CRC cell lines (22, 23). Mlecnik B et al. reported upregulation of ICAM-1 in CRC cells lowered the frequency of distant metastasis (23). The transfection of ICAM-1 into CRC cells inhibited tumor metastasis (27). Tachimori A et al. also demonstrated that liver metastases decreased in CRC cells expressing ICAM-1 compared with CRC cells not expressing ICAM-1 (65).

## Mechanisms of ICAM-1 in CRC metastasis

Metastasis of CRC is a complex process that is influenced by a variety of factors. Among these factors, ICAM-1 plays a key role, but the mechanisms of how ICAM-1 decreases metastasis of CRC are not completely clear. One potential mechanism is that ICAM-1 can activate the immune system to prevent metastasis of CRC (13). ICAM-1 promotes recognition and destruction of tumor cells by the immune cells (4). ICAM-1 increased lymphocytes recruitment, promoted lymphocytes to attach to CRC cells and lymphocyte-mediated tumor lysis, which may improve the immunosurveillance and restrict tumor metastasis (20, 24). It can also sensitize metastatic tumor cells to CTL-mediated killing and prevent tumor metastasis (66). A second mechanism may be that ICAM-1, as a morphogen, enhances tumor cells attachment to the extracellular matrix by promoting motility in the context of remodeling. Taglia L et al. showed that ICAM-1 mediated tumor cells attachment to the extracellular matrix and prevented tumour cells from detaching from the primary tumor and thus retarded metastasis (16). Thirdly, ST6GAL1 could mediate tumor metastasis by regulating the stability of ICAM-1 (22). It might increase ICAM-1 stability through sialylation and consequently inhibit CRC metastasis (67, 68). Fourthly, the mechanism may be due to GRP’s activation of the immune surveillance system (2, 69). FAK phosphorylation mediates GRP’s activation of the immune system and ICAM-1 is the downstream proteins of FAK pathway (58–63, 66–72).

## Soluble ICAM-1 in CRC

Apart from the membrane-bound ICAM-1 expressed on CRC cells, there exists a soluble form of ICAM-1 (sICAM-1) in serum. SICAM-1 was firstly identified in the serum of healthy volunteers by Seth et al, and its level is elevated in malignancies (73). Although the splice variant of sICAM-1 is truncated at the transmembrane domain, it retains all five extracellular Ig-like domains similarly to full-length ICAM-1 molecule, and its ability is conserved. In agreement with the small size of the transmembrane and cytoplasmic domains, sICAM-1 is only slightly smaller in size than its membrane-bound form.

The mechanism of sICAM-1 production is unclear, but it may be produced by proteolytic cleavage of membrane-bound ICAM-1, and released from the local cancer cell and enter the serum (73). Secondly, sICAM-1 is an inflammation-associated marker and is therefore increased in patients with an inflammatory TME. Thirdly, ICAM-1 rs5498 may have an effect on the levels of sICAM-1. Bielinski SJ et al reported the ICAM1 rs5498 G allele was associated with the level of sICAM-1 (74).

The level of sICAM-1 was elevated in CRC patients and can serve as a biomarker (**Table 1**) (15, 20, 75, 79, 80). The level of sICAM-1 was positively correlated with the tumor size, advanced stage and metastasis in CRC patients (9, 14, 15, 20, 75, 79–82). Basouglu et al. found that the serum sICAM-1 level was higher in CRC patients than that in the healthy controls and patients with advanced stage had higher sICAM-1 levels than those with a lower stage (81). Mantur et al. and Kang et al. also observed that CRC patients with higher sICAM-1 level were at a higher advanced stage (15, 75). The sICAM-1 level in patients with distant metastases increased compared with patients without metastases. High levels of sICAM-1 have been shown to be associated with liver metastasis in CRC (14, 15, 18). SICAM-1 levels decreased significantly after curative surgery for CRC (15).

**Table 1:** Comparison of sICAM-1 levels between patients and controls.

CRC patients	median age (years)	Gender (male:female)	sICAM-1 levels in patients (ng/mL)	controls	sICAM-1 levels in controls (ng/mL)	P value	References
40	NR	20:20	366.1±114.1	24	306.4±98.2	0.037	(15)
63	70	33:30	285.0	51	203	NR	(20)
56	57	32: 24	743.7±113.7*	25	345.7±49.8	P<0.001	(75)
297	67	185:112	266.5	40	242.7	NR	(76)
138	64	89:49	160.9 ±109.9	40	76.1 ±15.6	<0.001;	(77)
46	66	20:26	228.0±52.59	40	201.7±24.7	P<0.02	(78)

NR: not reported, *Dukes C and D.

Previous studies demonstrated that patients with higher sICAM-1 level revealed poor prognosis (14, 16, 23), while the patients with lower sICAM-1 level displayed an improved OS (83). Yamamoto Y et al. also reported high sICAM-1 level was associated with shorter OS in CRC patients treated with chemotherapy plus bevacizumab (84). Elevated sICAM-1 level is associated with a decreased OS and serves as independent prognostic biomarker, but the mechanisms are not completely clear. SICAM-1 can bind to circulating CTLs, inhibit the interaction between CTLs and tumour cells, and block immune recognition of tumor cells (85). It can also block NK cell-mediated toxicity and thus allow tumour cells to escape immune destruction (76). Moreover, it can promote angiogenesis and stimulate tumour cells growth (86). These findings are possible explanations for the poor prognosis.

## ICAM-1 single nucleotide polymorphisms in CRC

SNPs are the most common type of DNA sequence and analysis of ICAM-1 SNPs is important for studying the genetic features of CRC. Previous studies have suggested that ICAM-1 SNPs are associated with the risk of CRC.

### ICAM-1 SNPs and CRC risk

Several studies have assessed the relationship between CRC risk and ICAM-1 SNPs, but these results were controversial (**Table 2**). Some studies showed that ICAM-1 SNPs were associated with an increased CRC risk (5, 64, 87). George Theodoropoulos et al. firstly reported that ICAM-1 rs5498 was associated with an increasing CRC risk in CRC patients (87). Anbarasan C et al. and Wang QL et al. also reported ICAM-1 rs5498 increased the risk of CRC (5, 64). But in a meta-analysis, the ICAM-1 rs5498 decreased the risk of CRC in Caucasians (88). We found that ICAM-1 rs5498 was not correlated with the risk of CRC in Chinese CRC patients, but ICAM-1 rs5498 decreased the CRC risk in the subgroup of age≥61 (89). Ravindran Ankathil et al. found that ICAM-1 rs5498 did not show significant association for CRC risk in Malaysian CRC patients (90). In our previous study, ICAM-1 rs3093030 polymorphism did not influence CRC risk (89). For ICAM-1 rs179969 polymorphism, the frequencies of homozygous wild type was significantly higher in controls compared to CRC patients. The different findings may be due to different ethnicities, regions, ages or the limited sample sizes. In the future, an analysis of different SNPs may make it possible to describe the exact relations between polymorphisms and CRC risk.

**Table 2:** The relationship between CRC risk and ICAM-1 SNPs.

ICAM-1 SNPs	CRC patients	Gender (male:female)	median age(years)	Controls	CRC risk	ethnicities	References
rs5498	222	128:94	NR	200	increased	Greek population	(87)
rs5498	87	49:38	55.0	102	increased	Chinese population	(64)
rs5498	309	NR	NR	302	decreased	Caucasians	(88)
rs3093030	1003	620:383	61.1	1303	no significant difference	Chinese population	(89)
rs5498							
rs5498	280	140:140	53.2	280	no significant difference	Malaysian population	(90)
rs179969					increased		
rs5498	195	102:93	6.1	188	decreased	Chinese population	(91)

NR, not reported.

### ICAM-1 SNPs and tumor differentiation in CRC

ICAM-1 SNPs are correlated with differentiation of CRC. ICAM-1 rs5498 KK genotype in poorly differentiated patients was significantly higher than that in well- or moderately-differentiated patients, whereas ICAM-1 rs5498 KE+EE genotype in poorly-differentiated patients was lower than that in well- or moderately-differentiated patients. Wang QL also found that ICAM-1 rs5498 is significantly associated with well differentiation of CRC (64). Liu LB et al. reported ICAM-1 rs5498 was associated with the degree of tumor differentiation in the population of North China (91). The differentiation of CRC that correlates with ICAM-1 rs5498 may be of different ICAM-1 expression. ICAM-1 SNPs and multidrug resistance in CRC

MDR is one of the important factors leading to the failure of chemotherapy. Topo II and P-gp are MDR-associated protein and expression of them had a vital significance in chemotherapy for CRC. ICAM-1 rs5498 polymorphism was associated with MDR in CRC in a Chinese population (91). The high expression of Topo II and P-gp was observed in ICAM-1 rs5498 KK genotype, indicating that ICAM-1 rs5498 KK genotype might be associated with MDR in CRC (91).

## Anti-Tumor Therapy Targeting ICAM-1

Targeting ICAM-1 and its associated pathway might provide a new insight for treatments of CRC. However, ICAM-1 plays diverse roles in anti-tumor responses and immunity, therefore, the targeting treatments of ICAM-1 may be difficult. Blocking of ICAM-1 has been proven useful in rheumatoid arthritis (92), but targeting ICAM-1 in tumors have shown disappointing results.

Chimeric antigen receptor (CAR)-T cell therapy has shown remarkably effective in cancer treatment and ICAM-1 could be a promising target for CAR-T cells. Wei H et al. demonstrated ICAM1-specific CAR-T cells could recognize ICAM-1 expressing breast cancer cells and inhibit tumor growth *in vitro* and *in vivo (* 93), which provided a reference for CAR-T cell therapy in CRC. Further, CpG-ODN (oligodeoxynucleotides, ODN) vaccination caused up-regulation of ICAM-1 on tumor-associated blood vessel endothelia leading to tumor-infiltration of T cells and tumor suppression in mouse model of pancreatic carcinoma (94). Administer cytokines is the straight way to increase inflammatory signals, but this could lead to severe adverse events, so delivery of cytokines directly to the tumor site could reduce adverse events (95). Angiogenic factors in the TME can decrease ICAM-1 expression, so targeting angiogenesis could also increase the ICAM-1 expression (96). NF-κB pathway plays a central role in ICAM-1 expression, so blocking NF-κB pathway can inhibit the ICAM-1 expression. The acai polyphenolic extract inhibited the ICAM-1 expression by targeting NF-κB pathway (97). Flubendazole, the benzimidazole derivative used in the treatment of parasitic disease, suppressed the growth of colon cells by down-regulation of NF-κB and ICAM-1 (98). Perhaps, with a better understanding of the various functions of ICAM-1 and how their expression and function are regulated, the clinical value of ICAM-1 could be revisited for the improvement of therapeutic strategies.

## Conclusions and future perspectives

In this review, we provide the first comprehensive description of the knowledge regarding ICAM-1 in CRC. The pathogenesis of CRC involves various mechanisms, and ICAM-1 plays different roles. During cancer development, ICAM-1 mediates anti-tumor response including tumor antigen uptake, activation of tumor-specific T cells, leukocyte trafficking into the tumor site and tumor cell killing. ICAM-1 remains the focus of continued investigations and may serve as a promising prognostic biomarker, and a potential target for emerging therapies.

## Author contributions

GT and YZ provided direction and guidance throughout the preparation of this manuscript. ZQ and YW wrote and edited the manuscript. WT and YX reviewed and made significant revisions to the manuscript. RQ, ZZ and YP collected and prepared the related papers. All authors contributed to the article and approved the submitted version.

## Funding

This research was funded by “Liu Ge Yi Gong Cheng”of Jiangsu Province (Grant No. LGY2017022), Social Development Foundation of Zhenjiang (SH2019069) and Science Foundation of the Affiliated People’s Hospital of Jiangsu University (Y2019021-S)

## Conflict of interest

The authors declare that the research was conducted in the absence of any commercial or financial relationships that could be construed as a potential conflict of interest.

## Publisher’s note

All claims expressed in this article are solely those of the authors and do not necessarily represent those of their affiliated organizations, or those of the publisher, the editors and the reviewers. Any product that may be evaluated in this article, or claim that may be made by its manufacturer, is not guaranteed or endorsed by the publisher.
